# Evaluation of User’s Satisfaction With Orthotic and Prosthetic Devices and Services in Orthotics and Prosthetics Center of Iran University of Medical Sciences

**DOI:** 10.33137/cpoj.v5i1.37981

**Published:** 2022-07-17

**Authors:** A Baghbanbashi, B Farahmand, F Azadinia, M Jalali

**Affiliations:** Rehabilitation Research Center, Orthotics and Prosthetics Department, School of Rehabilitation Sciences, Iran University of Medical Sciences, Tehran, Iran.

**Keywords:** Orthosis, Prosthesis, satisfaction, Survey, Orthotics, Prosthetics, Rehabilitation

## Abstract

**BACKGROUND:**

The number of patients receiving orthotics and prosthetic services is increasing globally. A way to investigate patients’ insight about services provided to them is to evaluate their satisfaction with the received services. Furthermore, incorporating patients’ preferences into practice is an inseparable part of evidence-based practice. Applying such information in practice can contribute to the enhancement of the quality of services, the effectiveness of therapeutic interventions, and finally, the economic growth of service centers.

**OBJECTIVE(S):**

To evaluate patients’ satisfaction with the orthotic and prosthetic devices and services provided by the orthotics and prosthetics clinic of Iran University of Medical Sciences.

**METHODOLOGY:**

In this study, 173 people referring to the orthotics and prosthetics clinic of Iran University of Medical Sciences were recruited, and their satisfaction level was examined using the Orthotics and Prosthetics Users’ Survey questionnaire (OPUS) through a phone interview.

**FINDINGS:**

Concerning the devices, the mean value of total satisfaction score was 74:00±19.80 and the highest score belonged to no wear or rupture of the clothes with their devices (mean value = 4.76±0.84). In terms of services, the mean value of total satisfaction score was 72.12 ± 15.90 with the highest score belonging to the politeness of the clinic staff (mean value = 4.92±0.57). When the time point from receiving service was taken into account, the patients who received the service for less than a year showed higher satisfaction level with the service (p=0.024). Although satisfaction with the device was slightly higher among the participants who used the devices for more than a year, no significant difference was observed between the two groups in terms of device satisfaction.

**CONCLUSIONS:**

The overall satisfaction level from the devices and services was relatively high. However, the satisfaction level with the costs and coordination of the staff with the physicians showed a decline.

## INTRODUCTION

According to the World Health Organization (WHO) estimate in 2017 around 35–40 million people require prosthetic or orthotic services.^1^ Based on a report published by the state welfare organization of Iran, which supports the underprivileged, this organization provided orthotics and prosthetics services for about 47000 people between 2019 to 2021 in Iran.^2^ This is a small portion of those who need orthotics and prosthetics services in Iran. Many people receive services from rehabilitation centers of Red Crescent Society of Iran (provide rehabilitation services to more than 70000 people per year)^3^ or from private sector. The need for the evaluation of orthotic and prosthetic (O&P) services has recently increased.^4^ The assessment of the device and service quality is a prerequisite of the accreditation of the O&P centers.^5^ Regardless of the clinical practice ethics, outcome assessment can lead to the economic growth of the O&P facilities, as identification of the customers’ needs and willingness to satisfy them can preserve the customers for the organization.^6^ Evidence has shown a close relationship between customers’ satisfaction and the profitability of the organization.^7^ In this way, most organizations are interested in evaluating the quality of their service to improve their customers’ satisfaction and thereby survive the organization.^8^ The patient is a key factor in such assessments.^9^ Satisfaction with the services is a proper index for estimating the quality of services and their presentation to the clients, which helps adaptation of the service or product to the needs and expectations of the clients. Satisfaction is defined as the experience of the client after receiving a product or service.^10^ In other words, customer satisfaction and desirability of the product or service are related to the fulfillment of his/her needs. Satisfaction assessment in health management not only provides the information required to improve the health care services but also may indirectly improve the health state of individuals due to its positive psychological and mental effects. Patients’ satisfaction with health services is recognized by the World Health Organization (WHO) as one of the five indicators of service quality.^11,12^ Satisfaction assessment in the O&P field is more difficult than other parts of the health system because health practitioners, deliver wearable devices such as orthoses, prostheses, insoles, and medical shoes to the patients in addition to providing services such as patients assessment and training.^10,13^ This means that the satisfaction assessment should include two aspects: the service assessment and the quality assessment of the delivered device.^10^

DeRuyter et al.^14^ defined patients’ satisfaction along with other factors such as clinical outcomes, functional status, quality of life, and cost as key indicators in the field of assistive devices. Moreover, achieving more favorable clinical outcomes requires the patient’s adherence to the use of the prescribed orthoses or prostheses.^15,16^ The patient’s satisfaction is a factor playing a role in his/her adherence to the treatment.^17^ Some researchers believe that esthetic factors and convenience, as well as economic status and social issues, affect the level of satisfaction with an orthosis or a prosthesis and can improve patients’ adherence to the orthotic/prosthetic intervention.^18^ In Iran, the results of a survey on satisfaction with O&P facilities in a private clinic in 2012 showed that despite dissatisfaction with the appearance of device and its durability and delivery process, the patients expressed a high level of satisfaction with the fitness of their device.^19^

Various instruments and methods have been developed to measure the satisfaction of the users of orthotic and prosthetic devices among which, modified Servqual questionnaire,^4^ Quebec User Evaluation of Satisfaction with Assistive Technology,^20^ The Trinity Amputation and Prosthesis Experience Scales (TAPES),^21^ and orthotics and prosthetics users survey (OPUS)^22^ can be mentioned. The OPUS questionnaire designed by Heinemann et al.^22^ for estimating the satisfaction level of the users of orthotic and prosthetic devices and services has gained high validity and reliability.

Assessing the satisfaction level of users and identifying the factors that lead to dissatisfaction along with its possible solutions can provide valuable information to improve the quality of devices and services provided by O&P centers, which in turn can increase the client’s satisfaction, improve clients’ functional status, and finally advance the economic growth of these centers. Furthermore, as mentioned earlier, device and service quality assessment is a prerequisite in the accreditation of O&P facilities. Despite the significance of user satisfaction in the treatment success and economic growth of service provider centers, a limited number of studies have addressed this issue in the O&P field.^10,13,19,23,24^ Since accreditation is the responsibility of national universities of medical sciences of each province, the present study was conducted to investigate the satisfaction of patients referred to the O&P center of Iran University of medical sciences as an educational and therapy center.

## METHODOLOGY

The present study is descriptive-analytical research whose protocol was approved by the ethics committee at Iran University of Medical Sciences (IUMC). A total of 173 volunteers were selected among the people referring to the O&P center at the Rehabilitation School of Iran University of Medical Sciences through a convenience sampling method. Non-electronic (paper-based) data including patients’ contact information are stored in the O & P center. Patients’ contact information based on their permission were transmitted to the main investigator. The inclusion criterion was a history of at least 3 months of using orthoses or prostheses that were made and prescribed in the O&P center of the Rehabilitation school. Subjects who were unable to respond to the questionnaire or phone interview due to cognitive or speech problems were excluded from the study.

The origin version of OPUS questionnaire (satisfaction module) answered through phone interviews was used to assess the users’ satisfaction with the O&P devices and services.^22^ If the user was younger than ten, parents were contacted. The OPUS questionnaire includes 5 domains: lower extremity functional status (LEFS), upper extremity functional status (UEFS), client satisfaction with devices (CSD), client satisfaction with services (CSS), and Health-related quality of life (HRQoL). The validity and reliability of this questionnaire were confirmed in Persian (Cronbach’s alpha coefficient of 0.71 and 0.89 for device and service satisfaction, respectively).^25^

The satisfaction domain of the OPUS questionnaire is composed of 21 questions, 11 of which are related to the evaluation of “*satisfaction with the received device, i.e., orthoses and prostheses*” and the other 10 items are related to the evaluation of “*service satisfaction*”. The section related to “*device*” in the OPUS questionnaire explores various aspects of the patients’ satisfaction including proper fitting, weight, durability, maintenance, easy to put on, appearance, comfort of use, wear, and tear of clothing, pain-free when wearing the device, skin irritation and affordability of device repair and replacement.

The service section also assesses the patient’s satisfaction with the level of courtesy and respect of the center staff, staff response to concerns and questions of the participants, opportunities for the patient to express their concerns, training how to use the device, waiting time, patient involvement in the decision-making process, discussion of problems, receiving an appointment within a reasonable time, question the explanations to choose the most appropriate device, and coordination between staff of the center and therapists with the doctors. The scoring guideline was used to score the response to each question (5=Strongly Agree, 4= Agree, 3=Neither agree nor disagree, 2=Disagree,1=Strongly disagree). “*Satisfaction With Device*” score was the sum of the scores for items 1-11 (11 – 55). “*Satisfaction With Services*” score was the sum of the scores for items 12-21 (10 – 50). Higher scores indicate better outcomes for both measures. The provided table was used to convert the raw scores to Rasch measures (0-100).^26^

A demographic questionnaire was also completed which collected information on name, surname, age, gender, level of education, occupation, involved organ, involved side, cause of receiving the device, year of receiving the service, duration of device use, and the treatment costs (%) covered by insurance companies. The de-identified data has been stored in SPSS format and if requested, Excel format can be transformed. This data can be requested from the O & P department via Corresponding author.

### Statistical analysis:

To analyze the data, SPSS software (version 22) was used. Shapiro-Wilk test was used to ensure the normal distribution of overall satisfaction scores. Frequency was used to describe qualitative variables while mean and standard deviation were employed for quantitative variables. Kruskal-Wallis test was adopted to compare the satisfaction scores of the device and services between the age groups. A Mann-Whitney test was used to compare the two groups of men and women, as well as the groups who used the device for 3 months to a year and those who used the device for more than a year. The statistical significance level was set at 0.05.

## RESULTS

Men make up 58% of the study population whereas the percentage of women was 42%, the minimum and maximum age of clients were 4 and 85 years, respectively (**Table 1**). The mean and standard deviation of the users’ age was 32.1± 23.5 years. Most devices received by the clients were insoles and medical shoes (145 cases).

**Table 1:** Demographic characteristics and device types.

	N
**Gender**	100 Males, 73 Females
**Age (year)**	
<10	33
10-34	63
35-64	59
>65	18
**Device types**	
Footwear / insole	145 (Flatfoot/Cavus/Heel pain/Corn&Callus/Clubfoot/Leg length discrepancy/Diabetes)
Lower limb orthosis	20 (Stroke/CP/Neuropathy/Orthopedic)
Hallux valgus splint	8

### Satisfaction with Device:

The findings of this study indicated that in the device satisfaction section, the lowest level of satisfaction was related to the affordability to repair or replace the prosthesis or orthosis, as well as purchasing or maintaining them (**Table 2**). The higher satisfaction rate was for the item concerned with no wear or rupture of the clothes by the received device. In the present study, the overall satisfaction with the device was 74.00 ± 19.80. According to the results of a Kruskal-Wallis Test, satisfaction with the device (H (3) =0.97, p=0.808) did not exhibit a significant difference between age groups. A Mann-Whitney U test revealed no significant difference in the satisfaction level of males and females with a device (U=3132.0, z=-1.61, p=.107). Satisfaction with the device did not show a significant difference when participants were classified into two groups in terms of duration of using the device (3 months to 1 year and more than one year), albeit those who used the device for more than one year expressed higher satisfaction (U=1921, z=-1.52, p=.128).

**Table 2:** Mean, standard deviation and range of items and (device and service) total scores of the OPUS.

	Minimum	maximum	Mean ± SD
Fits well	0	5	4.62 ± 0.93
Manageable weight	0	5	4.42 ± 1.08
Comfortable	0	5	4.36 ± 1.14
Easy to put on	0	5	4.51 ± 1.04
Looks good	1	5	4.63 ± 0.84
Durable	1	5	4.72 ± 0.80
Wear and tear clothes	0	5	4.76 ± 0.84
Skin abrasion and irritation	1	5	4.55 ± 1.04
Pain free	1	5	4.49 ± 1.05
Afford purchase	0	5	3.95 ± 1.57
Afford repairs	0	5	3.95 ± 1.54
Appointment in reasonable time	0	5	4.77 ± 0.92
Showing courtesy	0	5	4.92 ± 0.57
Wait reasonable time	2	5	4.85 ± 0.54
Informed about choices	1	5	4.88 ± 0.53
Opportunity to express concerns	0	5	4.86 ± 0.58
Responsive to concerns	0	5	4.87 ± 0.53
Training for use and maintenance	1	5	4.87 ± 0.52
Discussion about problems	1	5	4.83 ± 0.66
Coordination with therapist	0	5	1.56 ± 2.11
Participation in decision making	0	5	4.46 ± 1.44
Satisfaction with device	33.06	100	74.00 ± 19.80
Satisfaction with service	37.72	100	72.12 ± 15.89

### Satisfaction with Service:

The satisfaction with the services summed up to 72.12 ±15.89. In terms of service satisfaction, the highest satisfaction was related to the courtesy and respectful behavior of the employees of the complex (4.92± 0.57). The lowest level of satisfaction from services was for the coordination of clinic staff with other treatment staff (1.56 ± 2.11). According to the results of a Kruskal-Wallis Test, satisfaction with services (H (3) =4.24, p=0.237) did not exhibit a significant difference between age groups. A Mann-Whitney U test revealed no significant difference in the satisfaction level of males and females with services (U=3594.0, z=-0.18, p=0.856). In terms of duration of the device use, service satisfaction was higher in the group that received the service for less than a year (**Table 3**). The two groups showed a significant difference in terms of service satisfaction (U=1756.5, z=-2.25, p=0.024).

**Table 3:** Comparison of level of satisfaction with device and with service between and among groups based on gender, age, and device use time.

Group	Satisfaction with device		Satisfaction with service	
**Age group** <10 10-34 35-64 >65	73.76 ± 20.71 72.45 ± 19.48 74.45 ± 19.47 78.37 ± 21.23	p=0.808	69.5 ± 14.43 74.59 ± 15.86 72.10 ± 17.68 68.35 ± 11.23	p=0.237
**Device use time** 3months-1year >1 year	69.74 ± 20.05 75.06 ± 19.66	p=0.128	79.86 ± 18.35 70.30 ± 14.74	p=0.024*
**Gender** Female Male	71.26 ± 20.42 75.99 ± 19.18	p=0.107	72.33 ± 16.39 71.97 ± 15.59	p=0.856

* Significantly different

## DISCUSSION

Despite the significance of user satisfaction in the treatment success and economic growth of service provider centers, a limited number of studies have addressed this issue in the O&P field.^10,13,19,23,24^ In this context, the present study aimed to assess the satisfaction of the clients of the O&P center of Iran University of medical sciences through OPUS questionnaire.

### Satisfaction with Device:

The findings of this study showed that in the device satisfaction section, the lowest level of satisfaction was related to the affordability to repair or replace the prosthesis or orthosis, followed by the affordability to purchase, and maintain them. The O&P center of the Rehabilitation School is an educational-clinical center and only the cost of materials and consumable parts are paid by the clients, so patients could obtain orthoses and prostheses much cheaper than private centers. Despite this privilege the “*affordability*” showed to be the main source of lower satisfaction in this survey. This survey was completed between 2015 and 2018. Based on the minimum monthly wage and benefits for a family with two children set by the government through these years, the cost of receiving lower limb orthoses in IUMS O&P center would be approximately 9%-12.4% of the minimum wage for foot orthoses, 19.7%-38.2% for medical shoes and 23.6%-36.5% for lower limb orthoses. Financial issues are one of the challenges in providing O&P services. Poor coverage of basic and supplementary insurance services makes the O&P users pay most of the costs out of pocket. Only a couple of Prostheses and Orthoses are covered by basic health insurance companies. Furthermore, although the general conditions of supplementary insurance services (private or group health) for P&O is better, many people find the insurance premiums high to afford. However, most of these companies set a ceiling to reimburse costs which may restrict the ability of patients for purchasing the services.

The most effective solution would be the boosted coverage of O&P services by basic insurance units.^27^ The policymakers of the health system should remove this barrier by enacting the necessary regulations. Iranian association of orthotics and prosthetics which is the main actor in this field is trying hard to convince them of the benefits of this policy.

In the study by Alsancak et al.^28^ and Ghoseiri et al.,^19^ the highest level of dissatisfaction was related to the appearance and esthetic aspects of the device. In the mentioned studies some of the participants received upper limb orthoses and prostheses. As upper limb orthosis and prostheses are more visible, it is logical that the appearance and aesthetic aspect of the device be the main concern and priority of participants. In the present study, insoles and medical shoes accounted for about 83% of the prescribed devices. Although desirable appearance is an important factor in patients’ adherence to O&P treatments, in the case of insoles and footwear, the comfort of the device, its weight, the quality of the material, and its effect on reducing symptoms may play more decisive roles in patients’ satisfaction. Based on the findings of the present study, the average satisfaction with the comfort of orthosis was 4.36 ± 1.14, which is higher than the report of Ghoseiri et al.^19^ (2.40 ± 1.00). Moreover, in the present study, the mean score of pain-free wearing of orthoses was 4.49 ± 1.05, reflecting the effectiveness of the devices prescribed in this center. However, in Ghoseiri et al.^19^ and Hoda et al.^23^ studies, this rate was 2.1± 0.9 and 3.39, respectively. In our study, the overall satisfaction of the device was 74.00 ±19.80, higher than the mentioned two studies (Ghoseiri et al.^19^: 46.6±15.2; and Hoda et al.^23^: 45.94 ± 11.62). However, in the research conducted by Bosmans et al.,^24^ the satisfaction rate was 78% among the clients of 15 O & P facilities in the Netherlands. Routhier et al.^29^ only assessed satisfaction with the myoelectric prosthesis in 18 patients with upper limb amputation which resulted in the satisfaction rate of 80%. It should be noted that deformity or specific neuromusculoskeletal conditions of clients may cause different psychological challenges, affecting their satisfaction with orthosis/prosthetic treatments.

### Satisfaction with Service:

Concerning service satisfaction, the mean total satisfaction score in this study was 72.10 ± 17.68, higher than Hoda et al.^23^ (65.77± 22.00) and Ghoseiri et al.^19^ (59.70 ± 12.00). The highest level of satisfaction was related to the politeness and proper respect of the employees of the complex with an average value of 4.92 ± 0.57. Similarly, Hoda et al.^23^ and Ghoseiri et al.^19^ reported this parameter with the highest level of satisfaction with respective average values of 74.40±4.00 and 3.30±0.70. The lowest level of satisfaction in the field of services in the present study was related to the coordination of clinic staff with other treatment staff with an average value of 1.56 ± 2.11, indicating the need for better communication and cooperation between rehabilitation physicians, physical therapists, and orthotists to achieve a successful rehabilitation treatment. The current study also revealed a higher level of satisfaction among the users who received services during the past year compared to those who received services for more than a year.

## CONCLUSION

The results of the present study indicated relatively high satisfaction with both the quality of orthopedic devices and services among patients referred to the O&P center of Iran University of Medical Sciences. However, when the costs associated with the device and the coordination of clinic staff with therapists and physicians were considered the satisfaction level declined. Assessment of clients’ satisfaction, as an approach to obtain their insights, can be a prominent part of evidence-based practices. Such information could highly contribute to improving the quality of services and upgrading the O&P facilities.

## DECLARATION OF CONFLICTING INTERESTS

The authors declare that they have no competing interests.

## AUTHORS CONTRIBUTION

**Ali Baghbanbashi**: Conceived the idea, designed the analysis, collected the data, contributed to data analysis, contributed to the final manuscript

**Behshid Farahmand**: Conceived the idea and designed the analysis, supervised the project, contributed to data analysis, discussed the results and contributed to the final manuscript

**Fatemeh Azadinia**: Conceived the idea and designed the analysis, performed the data analysis, took the lead in writing the manuscript

**Maryam Jalali**: Conceived the idea and designed the analysis, discussed the results, provided critical feedback and contributed to the final manuscript

## SOURCES OF SUPPORT

The authors would like to thank the Iran University of Medical Sciences for their official support of this study.

## ETHICAL APPROVAL

The present study is descriptive-analytical research whose protocol was approved by the ethics committee at Iran University of Medical Sciences (IUMC).
